# Feasibility and Acceptability of Delivering HIV Self‐Testing and Oral PrEP Refills to Female Sex Workers Through Community Drug Shops in Kampala, Uganda: A Single‐Arm Pilot Interventional Study

**DOI:** 10.1002/jia2.70220

**Published:** 2026-10-23

**Authors:** Catherine Nakaye, Denis Mawanda, Nelson Mukiza, Brenda Gati Mirembe, Rita Nakalega, Zubair Lukyamuzi, Hajira Kataike, Josephine Basirika, Clemensia Nakabiito, Mary Glenn Fowler, Renee Heffron

**Affiliations:** ^1^ Makerere University ‐ John Hopkins University Research Collaboration Kampala Uganda; ^2^ RineCynth Advisory Limited Kampala Uganda; ^3^ Kitebi Health Centre III Kampala Uganda; ^4^ Department of Pathology Johns Hopkins University Baltimore Maryland USA; ^5^ Department of Medicine University of Alabama at Birmingham Birmingham Alabama USA

**Keywords:** decentralization, drug shops, female sex workers, HIV, oral PrEP, self‐testing

## Abstract

**Introduction:**

Female sex workers (FSWs) in Uganda are disproportionately affected by HIV, with prevalence estimates reaching 37%. Decentralization of pre‐exposure prophylaxis (PrEP) delivery, including community‐based mechanisms, brings services closer to target populations. We assessed feasibility and acceptability of decentralized oral PrEP refill delivery with HIV self‐testing (HIVST) through a drug shop‐based model in Kampala, Uganda, among FSWs already using oral PrEP.

**Methods:**

From October 2024 to June 2025, we conducted a single‐arm pilot interventional study with 45 FSWs, aged≥18 years, receiving oral PrEP from a government facility for ≥ 1 month before enrolment. Participants were purposively linked to community drug shops convenient to their areas of residence for oral PrEP refills provision after HIVST at 1, 3 and 6 months. Feasibility was assessed by study participation rate, accrual and retention parameters. Structured questions using a 4‐point Likert scale were used to collect acceptability data at 3 and 6 months. Persistence was assessed by measuring proportions of participants who continued in care, received HIVST and oral PrEP refills from community drug shops. Descriptive analysis was conducted to summarize feasibility, acceptability and persistence to PrEP refills.

**Results:**

A total of 45 FSWs (median age [IQR]: 29 [26–35] years) were enrolled. All participants (100%, 45/45) willingly took up the community drug shop–based PrEP delivery model at baseline. Participant retention was 95.6%, 100% and 95.6% at 1, 3 and 6 months, respectively, which aligned with PrEP refill rates. Over 95% of FSWs at 3 and 6 months visits felt very comfortable and preferred receiving PrEP refills from community drug shops, with 100% of participants acknowledging them as very accessible, offering very private service and comfort to discuss sexual health with attending staff. HIVST convenient usability for drug shop staff possibly contributed to model acceptability.

**Conclusions:**

Community drug shop–based HIVST and oral PrEP refill delivery was feasible, acceptable and demonstrated high persistence with oral PrEP refills among this small FSW cohort in Uganda. These results warrant further research to evaluate the oral PrEP refill decentralization impact of this model when it is scaled up to reach a larger number of FSWs and other population groups.

## 1 Introduction

Female sex workers (FSWs) are a key population (KP) with high vulnerability to HIV acquisition in sub‐Saharan Africa (SSA), with an HIV incidence eight times higher than that of the global female population [1, 2, 3, 4]. Although significant strides have been made in delivering HIV prevention to FSWs, yielding a 50% reduction in new HIV acquisitions between 2010 and 2022 in some regions of SSA [2, 3, 5], HIV prevention services coverage, use and persistence, remains low globally [2]. In 2022, FSWs in Uganda had an estimated HIV prevalence of 31.3% and accounted for 11% of all new HIV acquisitions, the largest proportion experienced by a KP in the country, among whom low pre‐exposure prophylaxis (PrEP) accessibility has been cited as a primary driver of not using PrEP, and a missed opportunity to prevent new HIV acquisitions [6].

Oral PrEP and HIV self‐testing (HIVST) are complementary self‐controlled, evidence‐based HIV prevention tools that appeal to sex workers because of their effects on self‐efficacy and empowerment [7, 8], and are acceptable to FSWs in Uganda [9, 10, 11]. HIVST for HIV status determination is acceptable for community‐based PrEP refills and facilitates oral PrEP utilization [12, 13]. Although oral PrEP is highly effective and widely provided in Uganda through various strategies, including differentiated service delivery models, its use among FSWs is suboptimal due to inconvenient access and high transportation costs required to travel to facilities that provide PrEP [10, 14, 15]. High mobility, criminalization and stigma at HIV clinics are additional barriers to PrEP utilization among FSWs [14, 15, 16]. Research in Uganda indicates a preference and demand for PrEP services delivered within community settings rather than facility‐based services, due to greater accessibility and convenience, which may enhance PrEP persistence [17, 18].

Decentralization of PrEP service delivery for FSWs into communities offers discreetness, improves client‐centredness, de‐medicalizes services and circumvents facility‐level bottlenecks such as transportation costs, strict operating hours, long waiting times and limited privacy [8, 10, 14, 15, 19, 20, 21]. Previous studies in Uganda and similar settings found that community‐based PrEP decentralization models increase accessibility and enhance persistence in care compared to clinic‐based PrEP services among mobile populations like FSWs [18, 22]. Therefore, more innovative and pragmatic differentiated service delivery models leveraging community establishments as PrEP refill access points for FSWs already utilizing PrEP are needed. Community drug shops are private small retail outlets in Uganda licensed to sell the least harmful of controlled drugs (class C) and over‐the‐counter medications, differing from pharmacies which are licensed to sell all classes of drugs [23]. Community drug shops are accredited, authorized to operate and controlled by the Uganda National Drug Authority (NDA). These shops are conveniently located, easily accessible, offer quick and flexible health‐related services, and are highly trusted resources within communities [24]. Research in numerous low‐ and middle‐income countries, including Uganda, has shown that drug shops are important initial points of contact in communities for healthcare service provision [24, 25, 26]. Thus, community drug shops possess untapped potential for synergistic structured public−private partnerships of differentiated service delivery to bring HIV prevention service delivery like oral PrEP refills closer to target and most in need populations.

Community drug shops have not been previously explored in Uganda as a novel community‐based approach to further decentralize oral PrEP refill delivery among FSWs already using PrEP. We, therefore, assessed the feasibility and acceptability of utilizing community drug shops to provide HIVST and oral PrEP refills to FSWs and their persistence on oral PrEP refills when delivered in these settings in Kampala. Persistence was assessed as a co‐primary implementation outcome.

## 2 Methods

### 2.1 Study Design and Setting

We conducted a single‐arm pilot interventional study to assess feasibility and acceptability of community drug shop‐based HIVST and oral PrEP refill delivery decentralization among FSWs aged 18 years and older in Kampala, Uganda. Additionally, we assessed persistence with oral PrEP refill in the cohort. Study implementation was from October 2024 to June 2025 at Kitebi Health Centre (HC) III and three community drug shops within the centre's catchment area.

*Kitebi HC III* is a government‐owned facility under the Kampala Capital City Authority (KCCA) located in Rubaga Division of Kampala District, with a catchment population of over 410,400 people by 2024 [27]. The facility offers HIV services: HIV testing, treatment, care and prevention via distribution of condoms and provision of PrEP. At the time of the study, about 8400 clients accessed HIV care and prevention services, and the facility had established experience offering HIV prevention services to KP, with the majority of PrEP clients being FSWs. Kitebi HC III was specifically selected because it serves an area with one of the highest concentrations of hotspots for transactional sex, and Kitebi has one of the highest numbers of community drug shops in Kampala [24].

*Community drug shop‐based oral PrEP refill delivery*: This model was developed through a consultative community‐engaged approach. Community drug shops were initially approached to assess interest and commitment of their owners and staff in the delivery of oral PrEP refills to FSWs and collect data on shop eligibility. Drug shop inclusion criteria were: being located within the catchment area of Kitebi HC III; having an owner willing to allow study participation; possessing a valid, up‐to‐date license from NDA; and having qualified, licensed staff willing to undergo training on study intervention and PrEP delivery. Drug shop staff's qualifications included Clinical Officer, Pharmacy Technician/Dispenser, Registered/Enrolled Nurse, Registered/Enrolled Midwife or Comprehensive Nurse as mandated by NDA. Community drug shops meeting protocol‐specific inclusion criteria were selected to deliver HIVST and oral PrEP refill to participants at follow‐up visits.

### 2.2 Study Population and Recruitment

Community sensitization was conducted in high‐risk hotspots (brothels and bars) within the study area. The study population included FSWs residing or operating within Kitebi and receiving oral PrEP from Kitebi HC III. Potential participants were approached during their routine PrEP refill visits at the facility. Screening and enrolment procedures were conducted at Kitebi HC III, and potential participants were offered HIV testing as per the Uganda national HIV testing algorithm. Women were eligible to join the study if aged at least 18, engaged in sex work, had not acquired HIV and were using oral PrEP for at least 1 month with a plan for continued use.

### 2.3 Study Outcomes

The primary study outcomes were feasibility and acceptability of intervention, and persistence on oral PrEP refills during study follow‐up.

### 2.4 Sample Size Determination

A sample size between 24 and 50 is recommended for pilot feasibility studies [28], and study investigators determined that a sample of 45 FSWs was sufficient for the planned descriptive analysis. Three community drug shops were selected for the study, and each was logistically allocated 15 participants.

### 2.5 The Intervention

The study intervention included integrated delivery of HIVST and oral PrEP refills to participants at a community drug shop for 6 months from the date of study enrolment, with expected refills 1, 3 and 6 months after study initiation. Drug shop staff, two registered nurses and one enrolled midwife had an intensive 1‐day in‐person training on 20 September 2024 on PrEP delivery procedures encompassing HIV acquisition risk assessment, risk reduction counselling, sexually transmitted infections (STIs) syndromic assessments, performance and interpretation of HIVST, oral PrEP dispensation, counselling on PrEP adherence, side effects, gender‐based and intimate partner violence. Trainers included clinicians, a counsellor from Kitebi HC III PrEP Clinic, regulatory affairs coordinators, social scientists and a biostatistician. Refresher training was provided at community drug shops ahead of 1, 3 and 6 month participant visit periods. All study staff had training in Good Clinical Practice and human subjects’ protection. Drug shop staff training was conducted before participant recruitment began. Only enough HIVST kits, oral PrEP medication, condoms and lubricants stock as needed per follow‐up period were obtained from Kitebi HC III and transported to community drug shops ahead of participant visits. Study staff were in charge of obtaining study commodities from the facility.

### 2.6 Theoretical Framework: Consolidated Framework for Implementation Research

Consolidated Framework for Implementation Research (CFIR) underpinned the study's assessment of feasibility and acceptability, and guided evaluation of facilitators and barriers of the model [29, 30].

### 2.7 Study Procedures

At enrolment, participants were purposively assigned to a participating community drug shop in their area or close to their residence to receive oral PrEP refills at subsequent 1, 3 and 6 months. Linkage included being escorted by study staff to the assigned drug shop and introduction to drug shop staff to facilitate a warm handoff. The study team provided visit reminders about PrEP refill visits through phone calls, which are done as standard of care for PrEP users at Kitebi HC III.

At each follow‐up visit, the trained drug shop staff guided and supervised administration and use of OraQuick In‐Home HIV Test kits for participants. HIVST results were read and interpreted by drug shop staff by looking at the test cartridge after it was run.

At each follow‐up visit, oral PrEP was refilled for participants with a negative HIVST result. Participants were also offered condoms and lubricants, risk reduction counselling, oral PrEP adherence counselling, and referral to Kitebi HC III for diagnosis and management of suspected STIs. At the end of 6 months’ follow‐up, participants were referred back to Kitebi HC III for continued PrEP refill and other prevention services. Participants with positive HIVST results at drug shops were to be escorted by study staff to Kitebi HC III for HIV status confirmation, with pre‐test and post‐test HIV counselling performed according to national HIV testing services guiding principles [31]. Participants with confirmed HIV acquisition would be discontinued from study follow‐up and immediately referred to the facility antiretroviral therapy clinic.

### 2.8 Data Collection

A survey tool was used to assess the eligibility of community drug shops, their owner and staff. Participants’ duration on oral PrEP prior to study participation was confirmed from PrEP medical records at Kitebi HC III. An interviewer‐administered questionnaire was used to obtain information on participant demographic characteristics, condom use, contraceptive use, genital symptoms treatment and risk perception of HIV acquisition. Acceptability was assessed at 3 and 6 months using a structured interviewer‐administered questionnaire, and key domains included comfort with receiving PrEP refills at a drug shop, ease of access, privacy, comfort discussing sexual health with drug shop staff, preference for drug shop versus facility‐based service delivery, willingness to continue using model, willingness to recommend model to others and overall satisfaction with service delivery. Responses were measured using a 4‐point Likert scale ranging from “very comfortable to not comfortable” [32]. The acceptability questionnaire was developed by study investigators based on study objectives and underwent expert review for content validity; however, it was not formally validated or pilot‐tested before implementation. Completion of oral PrEP refill visit procedures was documented by drug shop staff in a study logbook. Potential sources of bias include selection bias, as participants were already using oral PrEP at enrolment. Additionally, acceptability outcomes may have been affected by courtesy and social desirability biases; and oral PrEP refill persistence may have been influenced by additional study support, including staff reminder calls.

### 2.9 Statistical Analysis

Data were analysed using STATA version 15 (StataCorp, College Station, TX, USA). Descriptive statistics were used to summarize participant characteristics, feasibility, acceptability and oral PrEP refill persistence outcomes. Continuous variables were summarized using medians and interquartile ranges (IQRs), as appropriate, while categorical variables were presented as frequencies and percentages. Feasibility was measured by recruitment and retention rates. Acceptability responses were summarized using frequencies and percentages. Missing data identified during data cleaning were handled using complete‐case analysis; no imputation was performed. Persistence was assessed by measuring the proportions of participants who continued in care and received oral PrEP refills at community drug shops at study visits. Given the feasibility study design and primarily descriptive objectives, confidence intervals, formal hypothesis testing, subgroup analyses and statistical comparisons between follow‐up time points were not performed.

### 2.10 Ethical Approval

The study protocol was approved by the Joint Clinical Research Centre (JCRC 2024–79) and Uganda National Council for Science and Technology (HS4556ES). Administrative clearance was obtained from KCCA. After having the study explained and their questions answered, written informed consent was obtained from all participants in English or Luganda. Illiterate participants provided informed consent in the presence of a literate impartial witness. Anonymity and confidentiality were strictly observed through use of participant identification numbers and limited access to participant records at community drug shops.

## 3 Results

### 3.1 Demographic Characteristics

A total of 55 potential participants were screened, and 45 were enrolled in the study. The median age was 29 (IQR: 26–35) years. The majority, 51.1% (23/45), had reached secondary education level, and most, 66.7% (30/45), had their sexual debut at ages 12−17 years (Table 1). The majority, 72.7% (32/44), had a primary sexual partner in the past 3 months prior to study entry, and most, 88.6% (39/44), had used a condom at their last vaginal sexual encounter. Nearly half, 47.7% (21/44), had sought treatment for genital symptoms in the prior 3 months. The majority, 79.6% (35/44), reported use of alcohol just before or during sex. Three‐quarters of participants perceived their HIV acquisition risk to be moderate. At the time of the study, more than half, 56.8% (25/44) of participants reported using a form of effective contraception, such as pills, implants or intrauterine devices.

**TABLE 1:** Demographic characteristics of female sex workers (FSWs) assigned to receive pre‐exposure prophylaxis (PrEP) refills at a drug shop.

Variable name	Frequency, *n* = 45	Percentage, %
Age of participant		
18−24 years	9	20
25 and above	36	80
Level of education completed		
None	2	4.4
Primary	20	44.4
Secondary level (completed/not)	23	51.1
Age at first sex		
12−17 years	30	66.7
18−22 years	15	33.3
Had a primary sexual partner at any time during the past 3 months	32	72.7%
Condom used during the last vaginal sex act	39	88.6%
Currently in a relationship with a partner who is living with HIV		
No	36	81.8%
Yes	1	2.3%
I don't know	7	15.9%
Had a drink containing alcohol just before or during sex in the past 30 days	35	79.6%
Used any recreational drugs in the past 3 months	13	29.6%
Received treatment for genital symptoms in the past 3 months	21	47.7%
Currently using any form of contraception (pills, injections, implants and intrauterine devices)	25	56.8%
Self‐perceived HIV risk		
High risk	3	6.8
Moderate risk	33	75
Low risk	8	18.2

### 3.2 Participant Beliefs on Oral PrEP Use, Disclosure and Social Support

At baseline, all participants (100%) strongly agreed that they were taking oral PrEP to protect themselves against HIV acquisition and believed that it is important to take it correctly and timely (Table 2). Almost all (44/45, 97.8%) understood that if oral PrEP was not taken as instructed, it may lead to HIV acquisition. Few participants (6.7%) had disclosed their PrEP use to family and friends, and 24.4% had disclosed to people they lived with.

**TABLE 2:** Participant beliefs on oral pre‐exposure prophylaxis (PrEP) use, disclosure and social support.

	Strongly agree *n* (%)	Agree *n* (%)	Neutral *n* (%)	Disagree *n* (%)	Strongly disagree *n* (%)
Oral PrEP usage and beliefs					
I am taking medication (oral PrEP) to protect against HIV.	45 (100.0)	0	0	0	0
It is important to take my oral PrEP correctly and on time.	45 (100.0)	0	0	0	0
If I don't take PrEP as instructed, I might get infected.	44 (97.8)	1 (2.2)	0	0	0
**Stigma and disclosure**					
Most of my family and friends know I am taking oral PrEP.	0	3 (6.7)	14 (31.1)	15 (33.3)	13 (28.9)
Most of the people I live with know that I am taking oral PrEP.	2 (4.4)	11 (24.4)	21 (46.7)	6 (13.3)	5 (11.1)
I shouldn't tell the people I live with that I am taking oral PrEP.	5 (11.1)	13 (28.9)	7 (15.6)	15 (33.3)	5 (11.1)
**Social support and housing/shelter**					
Household members who know I am on oral PrEP would help me remember to take my PrEP.	2 (4.4)	3 (6.7)	9 (20)	17 (37.8)	14 (31.1)
I feel supported by my family and friends when times are tough.	5 (11.1)	10 (22.2)	20 (44.4)	7 (15.6)	3 (6.7)
I feel like I have a stable place to live.	4 (8.9)	7 (15.6)	9 (20)	14 (31.1)	11 (24.4)
Sometimes, a homeless shelter is the only place I have to sleep.	20 (44.4)	18 (40)	2 (4.4)	0	5 (11.1)

Eleven percent (11.1%) of participants reported that household members who knew they were on oral PrEP would support them to remember to take their medications. When asked about emotional support, one‐third (33.3%) of participants felt supported by family and friends during tough times. Less than a quarter (24.5%) of participants felt that they had a stable place to live, 55.5% lacked a stable shelter and 84.4% reported that sometimes a homeless shelter was the only available place to sleep.

### 3.3 Feasibility of the Drug Shop‐Based Oral PrEP Refill Model and PrEP Persistence

The study participation rate was 81.8%. Participant retention was >95% during all follow‐up visits, with 95.6% FSWs returning to community drug shops to receive oral PrEP refills at month 1, 100% FSWs at month 3 and 95.6% FSWs at month 6 (Figure 1). All FSWs who returned to community drug shops chose to receive a refill of oral PrEP at the visit.

**FIGURE 1:**
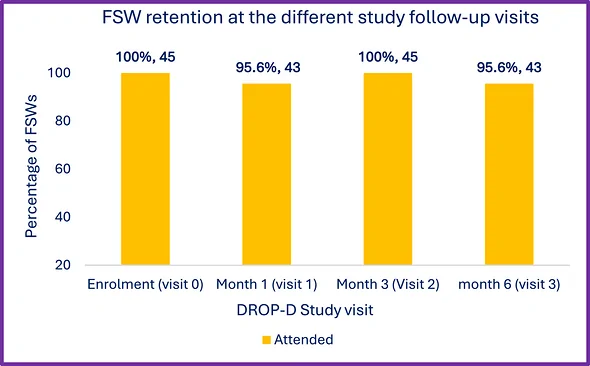
Visit attendance by female sex workers (FSWs) across four time points. (DROP‐D was the short study title acronym used).

### 3.4 Acceptability of the Drug Shop‐Based Oral PrEP Refill Model

At 3 and 6 months, over 95% of participants reported feeling very comfortable receiving oral PrEP refills from their assigned drug shop (95.6% at month 3 and 97.7% at month 6, Table 3). Similarly, ease of access was consistently affirmed, with 100% of participants reporting that it was “very easy” to receive oral PrEP refills at community drug shops during both visits. Furthermore, likelihood of continuing with this model remained high, with 86.7% and 95.3% of participants at 3 and 6 months, respectively, stating they were “very likely” to continue receiving oral PrEP refills if the service remained available at community drug shops. A strong preference for drug shop‐based over facility‐based oral PrEP refill delivery was expressed, with 93.3% at month 3 and 97.7% at month 6, favouring the drug shop setting. Participants also demonstrated high satisfaction with quality of service: 72.7% at month 3 and 93% at month 6 rated oral PrEP refill service delivery as “very good.” Regarding privacy, 93.3% at month 3 and 100% at month 6 felt the service was “very private.” Lastly, 100% of participants agreed that PrEP should be made available at community drug shops.

**TABLE 3:** Acceptability of oral pre‐exposure prophylaxis (PrEP) delivery and HIV self‐testing (HIVST) at assigned community drug shops.

Acceptability questions	Month 3, *n* = 45	Month 6, *n* = 43
Comfort level with receiving oral PrEP and HIVST from drug shop		
Comfortable	2 (4.4%)	1 (2.3%) 42 (97.7%)
Very comfortable	43 (95.6%)	
Comfort level with discussing sexual health and behaviour with drug shop staff		
Comfortable	7 (15.6%)	0 (0%) 0 (0%) 0 (0%) 43 (100%)
Not comfortable	3 (6.7%)	
Somewhat	3 (6.7%)	
Very comfortable	32 (71.1%)	
Degree of privacy of oral PrEP and HIVST service delivery at the drug shop		
Privacy	3 (6.7%)	0 (0%) 43 (100%)
Very private	42 (93.3%)	
Ease of receiving oral PrEP and HIVST refills at the drug shop		
Very easy	45 (100%)	43 (100%)
Preference for receipt of oral PrEP refills from a drug shop or a health facility		
I would prefer a drug shop	42 (93.3%)	42 (97.7%) 0 (0%) 1 (2.3%)
I would prefer a health facility	1 (2.2%)	
Neutral	2 (4.4%)	
Likelihood of continuing to receive oral PrEP refills if made available		
Very likely	39 (86.7%)	41 (95.3%) 2 (4.7%)
Likely	6 (13.3%)	
Likelihood of continuing to receive HIVST if made available		
Very likely	40 (88.9%)	42 (97.7%) 1 (2.3%)
Likely	5 (11.1%)	
Likelihood of recommending community drug shop delivery of oral PrEP and HIVST		
Somewhat likely	0 (0%)	1 (2.3%) 40 (93%) 2 (4.7%)
Very likely	36 (80%)	
Likely	9 (20%)	
Do you think that oral PrEP and HIVST should be made available at drug shops?		
Yes	45 (100%)	43 (100%)
How do you rate the oral PrEP service delivery by the drug shop?		
Good	12 (27.3%)	3 (7%) 40 (93%)
Very good	32 (72.7%)	
How do you rate the HIVST service delivery by the drug shop?		
Good	11 (24.4%)	4 (9.3%) 39 (90.7%)
Very good	34 (75.6%)	

## 4 Discussion

Pharmacy‐based PrEP delivery has been previously explored in a few studies in East Africa [33, 34, 35]. To our knowledge, this is the first study in Uganda to assess feasibility and acceptability of delivering HIVST and oral PrEP refills to FSWs through community drug shops. Our findings of high retention in care and continued oral PrEP refills through 6 months are very promising and suggest that this location could be explored further as a point of PrEP refills for FSWs already using PrEP. We observed oral PrEP refill continuation >95%. A PrEP continuation rate of 61.1% was found in a recent study conducted among FSWs accessing PrEP at a referral hospital in Uganda [18]. We also observed retention >95%. The observed retention in hospital delivery and community‐based delivery models described in a systematic review and meta‐analysis of HIV PrEP uptake, retention and adherence among FSWs in SSA was 83% [36]. The model was acceptable to participants, and over 95% felt very comfortable receiving oral PrEP refills from the assigned drug shop, which was consistently reported throughout the 6‐month follow‐up. Similarly, accessibility was high, with 100% of participants affirming that it was very easy to receive oral PrEP refills at community drug shops. Accessibility, convenience and flexibility in service hours at community drug shops with no queues, facilitating quick service for participants, possibly contributed to acceptability of the model, in contrast to facility‐based PrEP refill delivery where long queues and fixed clinic hours are a bottleneck [8, 10, 14, 15, 19]. HIVST possibly contributed to the acceptability of the model because of ease of use and convenience for healthcare providers in a community setting like drug shops.

Easy accessibility and good customer care are cited as some of the reasons why community drug shops are preferred for healthcare initiation [26]. Additionally, community drug shops gained community trust through their more convenient, quicker and flexible health services offered in comparison to other retail drug outlets like pharmacies, and they can provide flexible handling of clients with limited financial resources [25, 26]. Community drug shops have a smaller daily clientele volume of less than 25 clients, which facilitates short waiting times and quicker service delivery [24], and they provide sexual‐reproductive health‐related services, including provision of condoms and other contraceptives [25]. Thus, oral PrEP refill delivery at these establishments taps the potential of retail drug outlets to extend HIV prevention services within the community.

Data suggest that existing PrEP delivery models in SSA do not entirely address the inimitable challenges faced by FSWs while accessing PrEP, for example stigma, sex trade's high mobility nature and potential for violence from intimate partners; which challenges affect FSW PrEP use and retention in care [18, 36, 37, 38, 39]. There is an ongoing need to provide PrEP delivery models that address contextual population‐specific barriers of FSWs [36]. Our study findings, therefore, build on a wealth of research knowledge, which demonstrates the enormous significance of decentralization of PrEP service delivery from facilities to community settings like drop‐in centres in increasing community PrEP awareness, improving continuation on PrEP, as well as overcoming facility‐level barriers of PrEP service delivery like poor accessibility, high transportation costs, stigma, long waiting times and inflexible hours, among other barriers [10, 14, 15, 16, 38, 39]. Previous studies have already shown the significant association between accessibility of PrEP services and PrEP acceptance among FSWs [40].

Anticipated challenges of community drug shop oral PrEP refill delivery include concerns by drug shop owners and staff of disruption of routine drug shop business activities, reluctancy of some drug shop staff to undergo training on oral PrEP delivery and high staff turnover. To overcome the challenges, community drug shops should be assigned a manageable number of PrEP refill clients to avoid overcrowding and overwhelming the staff. Additionally, only drug shops with staff willing to be trained should be included in the model to ensure fidelity to oral PrEP delivery national guidelines. Lastly, close supervision is critical to provide refresher training routinely and identify and train new drug shop staff.

Strengths of the study include being among the first to engage community drug shops to deliver oral PrEP refills to FSWs in Kampala, Uganda. Additionally, use of HIVST by the FSWs is an avenue to foster self‐care in a community setting. Limitations of this pilot study include the absence of a comparison group, a small sample size both in the number of community drug shops and participants, and enrolment of existing PrEP using FSWs, which reduce its generalizability. Additionally, the study had a relatively short follow‐up period of 6 months and, therefore, long‐term oral PrEP usage was not evaluated. Furthermore, baseline model acceptability at month 1 and participant renal safety monitoring were not performed. Lastly, FSW oral PrEP adherence was not objectively measured with an intervention such as a point‐of‐care urine tenofovir test [41].

While acknowledging limitations and potential biases, the study findings are encouraging, and data can be used to inform future research utilizing community drug shops as potential locations to deliver oral PrEP refills for FSWs and other populations. These study findings can be used to support future research proposals such as stepped‐wedge cluster randomized trial designs and studies of cost‐effectiveness of the community drug shop oral PrEP refill delivery model.

## 5 Conclusions

In conclusion, our study found that community drug shop delivery of oral PrEP refills was feasible, acceptable and demonstrated high persistence with oral PrEP refills for FSWs in a Ugandan metropolitan setting, a model that could be further evaluated for its oral PrEP refill decentralization impact. Additional research is needed to objectively assess oral PrEP adherence at community drug shops during PrEP refill visits for FSWs.

## Author Contributions

CN, BGM, CN, MGF and RH designed the study. CN wrote the first draft of the manuscript. CN, DM, NM, BGM, RN, ZL, HK and JB participated in study implementation, while DM, NM and NC performed the statistical analyses. RH, CN, MGF and BGM participated in the study conception and interpretation of results. RH, CN and MGF supervised all stages of study implementation. All authors read and approved the final draft of the manuscript.

## Funding

Research reported in this publication was supported by the Fogarty International Center of the National Institutes of Health under Award Number D43TW009340, and RH was supported by K24MH123371.

## Conflicts of Interest

CN, DM, NM, BGM, RN, ZL, HK, JB, CN and MGF have nothing to disclose. RH has received donated medication from Gilead Sciences for research and has been a consultant to Merck, Sharp, and Dohme.

## Data Availability

The data that support the findings of this study are available from the corresponding author upon reasonable request through cnakaye@mujhu.org.
